# Difference in Aroma Components of Black Teas Processed on Different Dates in the Spring Season

**DOI:** 10.3390/foods12234368

**Published:** 2023-12-04

**Authors:** Penghui Yu, Yingjie Huang, Ziyi Li, Xi Zhao, Hao Huang, Ni Zhong, Hongfa Zheng, Qincao Chen

**Affiliations:** 1 Tea Research Institute, Hunan Academy of Agricultural Sciences, Changsha 410125, China; yphuihui@163.com (P.Y.); lzzx_11280609@163.com (X.Z.); haohuang_08@163.com (H.H.); daren_ni@163.com (N.Z.); 2 College of Agriculture, Jiangxi Agricultural University, Nanchang 330045, China; hyj605791@163.com (Y.H.); 18931077210@163.com (Z.L.)

**Keywords:** black tea, aroma, volatile compound, GC–MS, production date

## Abstract

Tea aroma greatly varies with the production date. This study investigated the aroma differences among black teas processed on different dates (March 23rd, April 8th, April 15th, April 27th, and May 7th) in the spring. A sensory evaluation showed that the black tea produced on April 15th had a strong and lasting sweet aroma and the highest score of 93.5. In total, 71 volatile compounds were identified, and alcohols were the predominant category, accounting for 60.98%. From March 23rd to May 7th, the total content of volatile compounds showed a parabolic change trend and reached its maximum on April 15th (715.27 μg/L); the flavor index first peaked on April 8th (23.25) and then gradually decreased. A multivariate statistical analysis showed that 39 volatile compounds were important, differential aroma components. An odor activity value (OAV) analysis showed that the predominant odorants were β-ionone, β-damascenone, linalool, (E)-β-ocimene, and geraniol, all with values larger than 100. The total OAVs of undesirable odorants decreased and reached their minimum (70.4) on April 27th, while the total OAVs of pleasant odorants and the ratio of pleasant/undesirable odorants showed inverse changes and reached their maximum (2182.1 and 31.0, respectively) on April 27th. Based on the significance of differences and OAVs, linalool, (E)-β-ocimene, geraniol, and (E,E)-2,4-nonadienal were considered as the key differential odorants. Combined with the sensory evaluation and the differences in aroma components, it was proposed that black teas produced around April 15th in the Hunan district are more likely to have a strong and lasting sweet aroma. This study will provide scientific guidance for the production of black tea in the Hunan district, China.

## 1. Introduction

Tea, processed from the tender shoots of tea plants (*Camellia sinensis* (L) O. Kuntze), is one of the most commonly consumed aromatic beverages, next to water, in the world. Black tea is one of six types of tea and accounts for approximately 75% of tea consumption globally [1]. In China, Congou black tea is a traditional black tea that has been processed for approximately 400 years and is preferred by local people compared to crush, tear, and curl (CTC) black tea, a type of black tea widely consumed in Western countries [2]. A consumer’s acceptability of a tea depends on its quality and price. The evaluation of tea quality includes the following five factors: appearance, taste, aroma, liquor color, and brewed leaves [3]. Congou black tea is famous for its brown and/or black color, sweet and mellow taste, sweet and/or floral aroma, and bright red liquor color [4].

A sweet scent is the basic aroma for black tea [5,6,7], and it can develop into a honey-like scent [8]; sweet potato-like scent [6]; or various floral and fruit scents, such as musky, malty, juicy–sweet, and rose-like odors [6,9,10]. Aroma is the ultimate manifestation of the interaction of various volatile components. It has been reported that aldehydes are the most abundant volatile compounds in black teas, followed by alcohols and esters [6,10]. In terms of individual volatile components, the main aroma components in black teas are hexanal, benzaldehyde, methyl salicylate, (E)-2-hexenal, benzene acetaldehyde, 3-methyl butanal, geraniol, linalool, benzyl alcohol, phenylethyl alcohol, cis-linalool oxide (furan), ethyl acetate, heptanal, and (E,E)-2,4-heptadienal, amongst others [6,10,11,12,13].

The volatile compounds in teas consist of volatiles present in fresh leaves and those generated during processing [5]. Thus, the volatile compounds in teas are greatly influenced by the tea cultivar [11,14], production season [15,16], altitude [17], shading treatment [14,18], manuring [19], processing method [3,20], etc. In terms of different production seasons, altitudes, and shading treatments, the climatic conditions, especially temperature and sunlight, are greatly disparate [21,22]. With different climatic conditions, the aroma components in fresh tea leaves vary greatly [21]. In addition, the metabolism of aroma precursors, including fatty acids [23], amino acids [24], and carotenoids [24,25], is significantly regulated by temperature and/or light. In China, tea is mainly manufactured in the spring season, i.e., from March to May. Although these three months all belong to spring, the climatic conditions are markedly different from March to May. For instance, the temperature becomes significantly higher and the sunlight substantially stronger from March to May. Therefore, in theory, the aroma qualities of teas in different production periods should be very different. Recent studies found that a production date in spring had a notable influence on the aroma quality of green teas [26,27].In addition, the production season showed a great influence on the aroma of black tea [15], green tea [16], and white tea [28]. However, the differences among black teas produced on different dates in the spring still remain unclear.

In the present study, the differences in aroma components among black teas processed on different dates in the spring were investigated via sensory evaluation and gas chromatography–mass spectrometry (GC–MS). The results of this study will enrich the aroma chemistry theory of black tea and be beneficial in guiding black tea production.

## 2. Materials and Methods

### 2.1. Chemicals and Reagents

Deionized water was produced using a Milli-Q water purification system (Millipore, Billerica, MA, USA). Ethyl caprate (99.0%) was obtained from the Sigma-Aldrich Corporation (Shanghai, China); NaCl was obtained from the J&K Scientific Corporation (Beijing, China).

### 2.2. Black Tea Samples

Clonal Zhuyeqi (ZYQ) (*Camellia sinensis* (L) O. Kuntze) tea plants selected for this study were planted in the experimental station of the Hunan Academy of Agricultural Sciences (113.4 E, 28.5 N; Changsha, China). To eliminate the influence of picking standards and weather conditions, all fresh leaves were plucked with a standard of one bud and one leaf on sunny days. The specific dates were March 23rd, April 8th, April 15th, April 27th, and May 7th in the spring of 2020. The manufacturing steps were as follows: approximately 3.5 kg of fresh tea leaves was firstly spread for indoor withering (22 °C, 50% humidity) until the moisture content reached approximately 62%; the withered tea leaves were then rolled for 70 min using a tea rolling machine (6CR-25, Sunyoung Machinery Ltd., Zhejiang, China); the rolled tea leaves were fermented at 30 °C with 90% humidity for 4 h in an environment-controlled cabinet (PRX-450D Guning Industrial Co., Ltd., Shanghai, China); lastly, the tea leaves were dried at 90 °C for 90 min using an aroma-enhancing machine (JY-6CHZ-7B Jiayou Machinery Co., Ltd., Fujian, China). The black teas produced on different dates were successively named ZYQ-1, ZYQ-2, ZYQ-3, ZYQ-4, and ZYQ-5, respectively.

### 2.3. Sensory Evaluation of Black Tea Samples

Sensory evaluation was carried out according to the Chinese national standards of the “Methodology of sensory evaluation of tea” (GB/T 23376–2018) and “Tea vocabulary for sensory evaluation” (GB/T 14487–2017). To ensure the impartiality and reliability of the sensory evaluation results, the black tea samples were coded before evaluating. The evaluation procedures were as follows: non-powdered tea samples (3.0 g) were brewed with 150 mL boiled water in a specialized cup; after brewing for 5.0 min, tea infusions were transferred to a specialized bowl; lastly, the aroma was descriptively evaluated by sniffing the brewed teas when the cup temperature was hot, mild, and cold. Each tea sample was independently evaluated by three experienced assessors, and the average score was used as the final score.

### 2.4. Extraction and Determination of Volatile Compounds

Volatile compounds were extracted via a method referred to in our previous study, with slight adjustments [29]. Tea powders (5.0 g) were firstly placed in a 100 mL glass vial, adding 10 µL ethyl caprate (100 mg/L) as an internal standard (IS). After adding 50 mL boiling deionized water, the vial was immediately sealed with a cover and equilibrated for 5.0 min at 60 °C. After this, a DVB/CAR/PDM coating fiber (50/30 µm; 1 cm; Supelco, Inc., Bellefonte, PA, USA) was inserted into the vial to adsorb the volatile compounds for 50 min. Finally, the volatile compounds were desorbed at 240 °C for 3 min for GC–MS analysis.

GC–MS analysis was carried out using a GC system (7890A, Agilent Technologies, Santa Clara, CA, USA) coupled with an MS detector (5975C, Agilent Technologies, Santa Clara, CA, USA). The column was a HP-5MS column (30 m × 250 µm × 0.25 µm, Agilent Technologies; Santa Clara, CA, USA), and the carrier gas was helium (99.999%) with a constant flow of 1.0 mL/min. The GC temperature raising procedures were as follows: 50 °C held for 5 min, slowly increased to 180 °C at 3 °C/min, and then increased to 250 °C at 10 °C/min and held for 3 min. The splitless injection mode was applied. The MS conditions were as follows: the ionization mode was EI; the ion source temperature was 230 °C; the quadrupole temperature was 150 °C; the mass scan scope was 50–550 *m*/*z*; and the electron impact ionization was −70 eV.

The concentrations of volatiles were calculated using the following formula: the concentration (µg/L) = (peak area of volatile/peak area of IS) × 10 µL × 100 mg/L.

### 2.5. Calculation of OAVs

The odor activity values (OAVs) of volatile compounds were calculated according to the formula of OAV = C/OT, in which C is the concentration of a volatile compound in tea and OT is its odor threshold in water. Volatile compounds with an OAV > 1 are generally considered to have a contribution to the aroma characteristics of tea [30].

### 2.6. Data Processing

Principal component analysis (PCA) using Pareto mode and partial least squares discriminant analysis (PLS-DA) using UV mode were performed using the Simca-P 13.0 software (Umetrics AB, Umea, Sweden). Variable importance for projection (VIP) values were calculated using PLS-DA. The significant differences were calculated by one-way analysis of variance (ANOVA) using the PASW Statistics software (Version 18.0, Chicago, IL, USA). The heat-map was generated by MultiExperiment Viewer 4.8.1 (Oracle Corporation, Redwood, CA, USA) after the data were UV-scaled.

## 3. Results and Discussion

### 3.1. Effect of Production Date on Black Tea Aroma Quality

As shown in Table 1, all five black teas emitted a sweet aroma. However, the intensity and endurance of the sweet aroma were different among the black teas. The intensity of the sweet aroma increased from ZYQ-1 (produced at March 10) to ZYQ-3 (produced at April 8th) and then slightly decreased in ZYQ-4 (produced at April 27th) and ZYQ-5 (produced at May 7th). In addition, ZYQ-3 had the most long-lasting sweet aroma, followed by ZYQ-2 and ZYQ-5. In particular, ZYQ-4 presented a slight floral scent. In total, ZYQ-3 had the best aroma quality and the highest score, followed by ZYQ-2 and ZYQ-5, ZYQ-4, and ZYQ-1.

### 3.2. Identification and Integral Comparison of Volatile Compounds

To further illuminate the differences in the aroma components in black teas processed on different dates in the spring season, the volatile compounds were determined using GC–MS. In this study, a total of 71 volatile compounds were identified, consisting of 7 aromatic hydrocarbons, 7 alkenes, 18 alcohols, 21 aldehydes, 7 ketones, 8 esters, 2 heterocyclic compounds, and 1 sulfur compound (Table 2). Alcohols were the predominant category, reaching an average of 388.20 μg/L and accounting for 60.98%, followed by esters (119.11 μg/L, 18.63%) and aldehydes (70.35 μg/L, 11.36%) (Table 2 and Figure S1). This is not consistent with previous studies [6,10]. This discrepancy may be due to the differences in the detection method. Heterocyclic compounds were the least abundant aroma components (Table 2 and Figure S1). In terms of individual volatile compounds, according to their average content, geraniol was the most abundant volatile, followed by methyl salicylate, linalool, phenylethyl alcohol, trans-linalool oxide (furanoid), benzene acetaldehyde, myrcene, nonanal, nerol, nerolidol, cis-linalool oxide (furanoid), and geranial, etc., which is essentially consistent with the results of previous studies [6,10,11,12,13].

**Figure 1:**
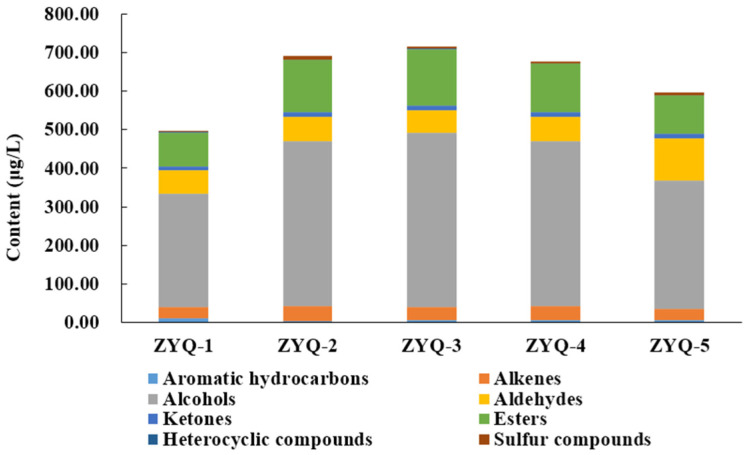
The content of different types of volatile compounds in black tea samples.

As shown in Table 2 and Figure 1, there were obvious differences in the content of different types of volatile compounds. The total content of volatile compounds first increased and then gradually decreased. Black teas produced on April 15th (ZYQ-3) and March 23rd (ZYQ-1) had the highest and lowest total content, respectively. It was reported that black tea produced in autumn had the highest content of volatile compounds, successively followed by that produced in spring and summer [15]. This implies that an appropriate temperature is conducive to the accumulation of aroma components and their precursors in fresh tea leaves, thus promoting the aroma concentration of black tea, while an excessively high temperature may bring the opposite effect. From March 23rd to May 7th, the temperature continues to increase. From the perspective of the total content of volatile compounds, the most suitable temperature to achieve a good black tea aroma is around April 15th in the Hunan district, China. The changes in the content of alkenes, alcohols, and esters were consistent with that in the total content of volatile compounds. The content of aldehydes was dramatically increased on May 7th (ZYQ-5). The content of ketones and sulfur compounds displayed an increasing trend, while the content of aromatic hydrocarbons and heterocyclic compounds showed irregular change trends.

**Figure 2:**
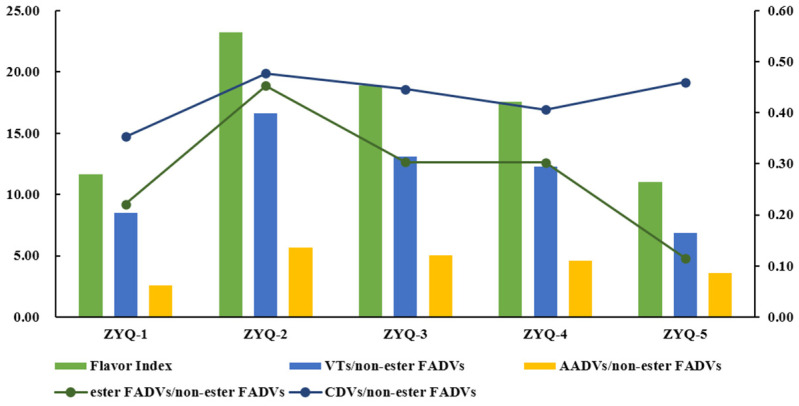
The changes in flavor index of various types of volatile compounds.

According to the synthetic pathways, volatile compounds can be divided into fatty-acid-derived volatiles (FADVs), amino-acid-derived volatiles (AADVs), volatile terpenoids (VTs), and carotenoid-derived volatiles (CDVs) [35]. In this study, 25 FADVs, 8 AADVs, 20 VTs, and 7 CDVs were detected (Table 2). These volatile compounds can be divided into two groups on the basis of their odor characteristics: Group I compounds mainly comprise alcoholic, aldehydic, and keto FADVs (non-ester FADVs), which impart an undesirable green and/or grassy odor to black tea; Group II compounds mainly comprise AADVs, VTs, and CDVs, which impart a sweet and/or flowery odor to black tea [36]. Unlike non-ester FADVs, most ester FADVs in teas generally emit pleasant floral, fruity, and fresh odors [37]. Thus, ester FADVs can be classified as Group II compounds. The ratio of the sum content of Group II compounds to that of Group I compounds is known as the flavor index, which positively reflects the flavor of black tea [36]. As shown in Figure 2 and Table S1, the total flavor index was first greatly increased and then largely decreased, peaking in ZYQ-2. The individual flavor indexes of VTs, AADVs, CDVs, and ester FADVs displayed a similar change trend to that of the total flavor index. In terms of ZYQ-2 and ZYQ-3, the increasing intensity in the flavor index of AADVs and ester FADVs was higher than that of VTs and CDVs (Table S2). Thus, AADVs and ester FADVs made larger contributions to the improvement in the aroma quality of the ZYQ-2 and ZYQ-3 black teas. In total, from the perspective of the flavor index, ZYQ-2 black tea would have the best aroma quality, followed by ZYQ-3, which is essentially in line with the sensory evaluation results (Table 1).

### 3.3. Effect of Production Date on the Individual Aroma Components of Black Teas

**Figure 3:**
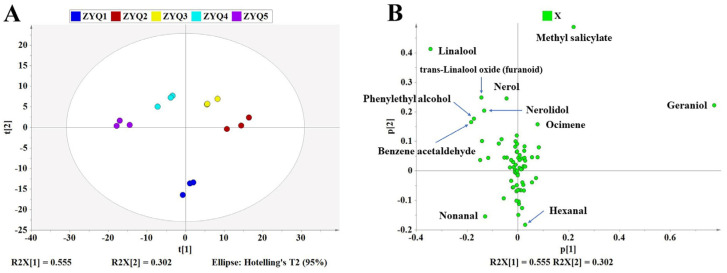
PCA score and loading plot of black tea samples. (**A**): PCA score plot; (**B**): PCA loading plot. R^2^X = 0.857, Q^2^ = 0.658. The data were Pareto-scaled.

PCA was first performed to determine the integral differences in volatile compounds and screen the main volatile compounds distinguishing the black teas. As shown in Figure 3A, ZYQ-1 was completely separated from the other black teas in the PC2 direction, while ZYQ-2, ZYQ-3, ZYQ-4, and ZYQ-5 were successively separated from each other in the PC1 direction. To further screen the most important volatile compounds distinguishing black tea samples produced on different dates, loading plots were generated. As shown in Figure 3B, the volatile components that greatly contributed to the differences among the black teas were geraniol, methyl salicylate, linalool, trans-linalool oxide, nerol, nerolidol, hexanal, phenylethyl alcohol, benzene acetaldehyde, nonanal, etc. They were all previously reported as important aroma-active compounds in black teas [8,10,11,38]. It was interesting that the Group I volatiles were separated from the Group II volatiles by PC2.

**Figure 4:**
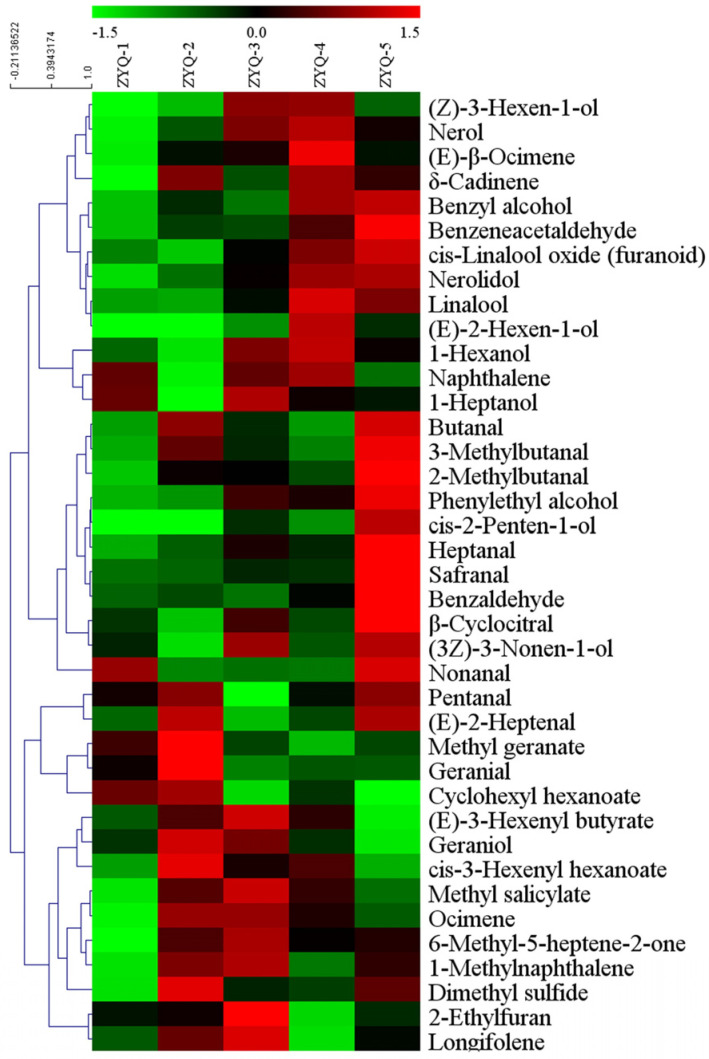
Heat-map of the levels of differential volatile compounds. The data were UV-scaled and clustered according to Pearson correlation coefficients.

To better understand the differences in the aroma components among the black teas produced on different dates in the spring season, important differential volatile compounds were screened based on ANOVA (*p* < 0.05) and PLS-DA (VIP > 1). In total, 39 important differential volatile compounds were identified (Table 2 and Figure 4). It seemed that there were no obvious change patterns in the content of volatile compounds. However, differential volatile compounds roughly presented three distribution patterns (Figure 4). The content of (Z)-3-hexen-1-ol, nerol, (E)-β-ocimene, δ-cadinene, benzyl alcohol, benzene acetaldehyde, cis-linalool oxide (furanoid), nerolidol, linalool, (E)-2-hexen-1-ol, and 1-hexanol showed an increasing trend from ZYQ-1 to ZYQ-5 and reached the maximum in ZYQ-4 or ZYQ-5. Nerol, (E)-β-ocimene, δ-cadinene, benzyl alcohol, benzene acetaldehyde, cis-linalool oxide (furanoid), nerolidol, and linalool are VTs and AADVs [5] and generally present pleasant odors [36]. (Z)-3-hexen-1-ol, (E)-2-hexen-1-ol, and 1-hexanol are FADVs [5] and mainly present unpleasant odors in black teas [36]. The content of butanal, 3-methylbutanal, 2-methylbutanal, phenylethyl alcohol, cis-2-penten-1-ol, heptanal, safranal, benzaldehyde, and β-cyclocitral, etc., showed a significant increase in ZYQ-5. Safranal and β-cyclocitral are derived from the degradation of carotenoids [7]. The significant increase in their content resulted in the raising of the flavor index of CDVs in ZYQ-5 (Figure 2). Other volatile compounds are FADVs and AADVs [5]. (E)-2-heptenal, pentanal, methyl geranate, geranial, cyclohexyl hexanoate, (E)-3-hexenyl butyrate, geraniol, cis-3-hexenyl hexanoate, methyl salicylate, ocimene, 6-methyl-5-heptene-2-one, and longifolene showed the highest content in ZYQ-2 and ZYQ-3. Geraniol is the key odorant responsible for the characteristic aroma of Keemun black tea [10,20], and it is the most abundant aroma component [20,39]. In this study, geraniol was also the most abundant volatile and showed the highest content in ZYQ-2 (Table 2), which was in line with the fact that the total and VT flavor index were the highest in ZYQ-2 (Figure 2).

### 3.4. Screening of the Key Differential Odorants Based on OAV

The contributions of individual volatile compounds to the overall aroma are closely related to their OT in addition to their concentrations. Volatile compounds with low concentrations frequently make a larger contribution to the tea aroma than those with high concentrations [40]. Compared with the concentration, the OAV provides a more reasonable evaluation of individual aroma components’ contributions to the overall flavor. Thus, it has been extensively applied in flavor research. As shown in Table 3, the OAVs of 25 aroma compounds were greater than 1 in at least one black tea. The total OAV of odorants was gradually increased from 1762.1 (ZYQ-1) to 2252.5 (ZYQ-4), and then slightly decreased to 2055.1 (ZYQ-5), which was consistent with the changes in the content of volatile compounds (Figure 1). The predominant odorants were β-ionone, β-damascenone, linalool, (E)-β-ocimene, and geraniol, all larger than 100. Owing to their high OAVs, they were considered as the key aroma-active compounds in ZYQ black teas. β-Ionone, β-damascenone, linalool, and geraniol have been previously identified as important aroma-active compounds in black teas [8,10,11,13,41], while (E)-β-ocimene was first identified as an important aroma-active compound for black teas. In addition, (E,E)-2,4-nonadienal, cis-3-hexenyl isovalerate, and (E)-2-nonenal showed moderate OAVs. The OAVs of naphthalene, ocimene, δ-cadinene, cis-linalool oxide (furanoid), trans-linalool oxide (furanoid), 3-methylbutanal, 2-methylbutanal, and benzene acetaldehyde, etc., were lower than 10; thus, these odorants may have comparatively smaller contributions to the aroma of ZYQ black teas.

With the exception of β-damascenone, β-ionone, and cis-3-hexenyl isovalerate, the other 22 odorants showed significant differences in their concentrations (Table 2). As linalool, (E)-β-ocimene, geraniol, and (E,E)-2,4-nonadienal showed much higher OAVs compared with other differential odorants, they were deemed the key differential odorants affecting the aroma quality of the black teas produced on different dates in the spring season. Linalool, (E)-β-ocimene, and geraniol are VTs (Table 1) and pleasant odorants emitting floral, sweet, and warm scents [7,32,33]. It is worth noting that the OAVs of linalool, (E)-β-ocimene, and β-damascenone reached the maximum in ZYQ-4, which supported the notion that ZYQ-4 presented a slight floral aroma (Table 1). (E,E)-2,4-nonadienal is an FADV emitting a cucumber-like scent [8]. In addition, (E)-2-nonenal, 3-methylbutanal, and nonanal showed moderate OAVs and thus were considered to have a considerable impact on the difference in aroma quality. Other odorants, such as ocimene, δ-cadinene, cis-linalool oxide (furanoid), and nerolidol, imposed a smaller influence.

According to the aroma characteristics, these odorants can be divided into two classes: Class I odorants mainly emit unpleasant scents, such as green, grassy, or pungent odors; Class II odorants mainly emit pleasant scents, such as floral, fruity, or sweet odors. The Class I odorants consisted of naphthalene, hexanal, (E)-2-octenal, nonanal, (E)-2-nonenal, (E,E)-2,4-nonadienal, and methyl salicylate; and the Class II odorants included β-ionone, β-damascenone, linalool, (E)-β-ocimene, geraniol, cis-3-hexenyl isovalerate, 3-methylbutanal, and benzene acetaldehyde, etc. The total OAV of Class I odorants gradually decreased from 145.1 (ZYQ-1) to 70.4 (ZYQ-4) and then increased to 101.1 (ZYQ-5), while the total OAV of Class II odorants showed an inverse change trend. In addition, the ratio of Group II/Group I OAVs was in line with the change in the total OAV of Class II odorants (Table 3).

In summary, combined with the sensory evaluation and the differences in the total content of volatile compounds, flavor index, and OAVs, it is proposed that black teas produced around April 15th will have relatively better aroma quality compared with those produced on other dates in the spring season (Table 1).

## 4. Conclusions

This study investigated the differences in the aroma quality and volatile components of black teas produced at different dates in the spring season. The sensory evaluation showed that the ZYQ-3 black tea (April 15th) had the best aroma quality. The predominant volatile compounds were geraniol, methyl salicylate, linalool, phenylethyl alcohol, and trans-linalool oxide (furanoid), etc. The total content of volatile compounds was obviously different among the black teas and reached the maximum in ZYQ-3 (April 15th). The flavor index showed the highest value in the ZYQ-2 black tea (April 8th). The OAV analysis showed that the predominant aroma-active compounds were β-ionone, β-damascenone, linalool, (E)-β-ocimene, and geraniol. The multivariate statistical analysis and OAV analysis implied that linalool, (E)-β-ocimene, geraniol, and (E,E)-2,4-nonadienal were the key differential odorants determining the aroma of the black teas. Combined with the sensory evaluation and the differences in aroma components, it is proposed that black teas produced on approximately April 15th are more likely to have better aroma quality. This study can provide scientific guidance for the production of black tea in the Hunan district, China.

## Figures and Tables

**Table 1:** The sensory evaluation of the aroma quality of black teas.

	ZYQ-1	ZYQ-2	ZYQ-3	ZYQ-4	ZYQ-5
Aroma description	Sweet, approaching strong	Sweet, strong and lasting	Sweet, strong and lasting	Sweet, nearly strong, with slight floral scent	Sweet, strong and lasting
Score	92.2	93.2	93.5	93.0	93.2

**Table 2:** Odor thresholds and the content (μg/L) of identified volatile compounds.

No.		Class	OT (μg/L)	ZYQ-1	ZYQ-2	ZYQ-3	ZYQ-4	ZYQ-5	*p*-Value	VIP
	Aromatic hydrocarbons			11.17 ± 1.67	4.87 ± 0.85	6.78 ± 0.57	5.86 ± 0.78	5.54 ± 0.52		
1	Methylbenzene		73 [31]	nd	0.47 ± 0.09	0.48 ± 0.05	0.40 ± 0.03	0.31 ± 0.06	0.034	0.91
2	Styrene			1.53 ± 0.13	0.50 ± 0.07	0.76 ± 0.11	0.55 ± 0.14	0.72 ± 0.10	0.000	0.89
3	Naphthalene		0.44 [32]	2.73 ± 0.64	1.19 ± 0.67	2.73 ± 0.37	3.00 ± 0.63	1.79 ± 0.33	0.011	1.19
4	1-Methylnaphthalene		8 [33]	0.49 ± 0.11	1.11 ± 0.16	1.20 ± 0.21	0.67 ± 0.09	0.97 ± 0.15	0.001	1.27
5	2-Methylnaphthalene			1.47 ± 0.53	0.56 ± 0.12	0.62 ± 0.03	0.40 ± 0.12	0.48 ± 0.04	0.002	0.78
6	2,6-Di-tert-butylbenzoquinone			4.94 ± 0.59	1.04 ± 0.19	0.98 ± 0.11	0.84 ± 0.10	1.27 ± 0.04	0.000	0.86
	Alkenes			28.41 ± 3.08	37.79 ± 2.83	32.87 ± 1.48	37.3 ± 4.34	28.56 ± 1.51		
7	Cubenene	VT		4.25 ± 0.62	1.71 ± 0.32	1.40 ± 0.04	2.03 ± 0.29	1.55 ± 0.15	0.000	0.93
8	Myrcene	VT	15 [3,10]	9.18 ± 1.24	11.75 ± 1.15	9.62 ± 0.39	10.71 ± 1.17	8.22 ± 0.46	0.009	0.99
9	Limonene	VT	10 [10]	5.74 ± 0.61	2.90 ± 0.38	2.55 ± 0.11	2.86 ± 0.36	2.35 ± 0.40	0.000	0.87
10	(E)-β-Ocimene	VT	0.02 [34]	2.31 ± 0.34	4.13 ± 0.66	4.47 ± 0.67	6.24 ± 0.90	4.11 ± 0.77	0.001	1.16
11	Ocimene	VT	6.7 [15]	2.64 ± 0.68	8.65 ± 0.49	8.65 ± 0.49	6.86 ± 0.54	5.01 ± 0.68	0.000	1.06
12	α-Cubebene	VT	14 [20]	1.96 ± 0.25	2.19 ± 0.39	1.58 ± 0.18	2.39 ± 0.40	1.82 ± 0.36	0.088	1.19
13	Longifolene	VT	2 [15]	0.84 ± 0.30	1.15 ± 0.23	1.35 ± 0.24	0.61 ± 0.06	0.97 ± 0.21	0.020	1.38
14	δ-Cadinene	VT	1.5 [32]	1.50 ± 0.18	5.31 ± 0.45	3.24 ± 0.15	5.61 ± 1.07	4.54 ± 0.84	0.000	1.06
	Alcohols			295.55 ± 19.71	428.25 ± 18.14	453.85 ± 25.81	428.22 ± 18.98	335.15 ± 8.38		
15	cis-2-Penten-1-ol	FADV	720 [33]	nd	nd	0.43 ± 0.07	0.25 ± 0.06	0.84 ± 0.14	0.001	1.07
16	(Z)-3-Hexen-1-ol	FADV	13 [7]	nd	1.64 ± 0.08	2.46 ± 0.42	2.49 ± 0.37	1.87 ± 0.29	0.025	1.04
17	(E)-2-Hexen-1-ol	FADV	100 [33]	nd	nd	0.54 ± 0.07	1.55 ± 0.19	0.86 ± 0.06	0.000	1.28
18	1-Hexanol	FADV	5.6 [33]	0.85 ± 0.17	0.68 ± 0.06	1.15 ± 0.16	1.24 ± 0.07	1.00 ± 0.19	0.005	1.15
19	1-Heptanol	FADV	3 [31]	0.63 ± 0.05	0.24 ± 0.02	0.71 ± 0.04	0.54 ± 0.07	0.50 ± 0.08	0.000	1.32
20	1-Octen-3-ol	FADV	1.5 [31]	0.18 ± 0.01	0.57 ± 0.10	0.51 ± 0.02	0.63 ± 0.10	0.46 ± 0.06	0.000	0.88
21	Benzyl alcohol	AADV	100 [3,31]	0.71 ± 0.13	1.19 ± 0.31	0.95 ± 0.29	1.82 ± 0.04	1.92 ± 0.75	0.013	1.02
22	cis-Linalool oxide (furanoid)	VT	6 [15]	5.49 ± 0.33	4.10 ± 0.35	7.98 ± 0.76	10.51 ± 0.94	12.06 ± 1.12	0.000	1.11
23	trans-Linalool oxide (furanoid)	VT	6 [15]	5.40 ± 0.07	10.57 ± 1.03	19.66 ± 1.43	18.93 ± 1.64	23.71 ± 1.59	0.000	0.96
24	Linalool	VT	0.22 [33]	62.61 ± 4.66	61.25 ± 3.81	88.78 ± 5.32	129.12 ± 4.66	112.35 ± 8.74	0.000	1.22
25	Phenylethyl alcohol	AADV	60 [31]	10.63 ± 0.52	11.88 ± 0.67	20.59 ± 1.73	19.15 ± 1.06	27.74 ± 2.98	0.000	1.01
26	(E)-Pinocarveol	VT		1.07 ± 0.29	0.78 ± 0.09	0.70 ± 0.13	0.34 ± 0.07	0.51 ± 0.12	0.002	0.97
27	(Z)-3-Nonen-1-ol	FADV		1.14 ± 0.19	0.67 ± 0.14	1.61 ± 0.05	1.01 ± 0.04	1.67 ± 0.45	0.001	1.20
28	1-Nonanol	FADV	45.5 [33]	nd	nd	5.51 ± 1.07	5.74 ± 0.40	4.92 ± 0.83	0.487	0.52
29	Nerol	VT	49 [33]	2.49 ± 0.52	7.03 ± 0.54	13.03 ± 0.11	14.74 ± 1.41	10.02 ± 0.82	0.000	1.04
30	Geraniol	VT	3.2 [7]	202.59 ± 15.1	320.11 ± 20.68	278.79 ± 21.78	204.32 ± 16.56	119.36 ± 6.8	0.000	1.20
31	Nerolidol	VT	10 [20,31]	1.79 ± 0.54	5.11 ± 0.88	8.86 ± 0.68	13.45 ± 1.01	13.92 ± 1.73	0.000	1.01
32	Epicubenol	VT		nd	2.43 ± 0.65	1.58 ± 0.24	2.40 ± 0.56	1.43 ± 0.26	0.055	0.97
	Aldehydes			59.2 ± 3.65	63.72 ± 1.32	57.00 ± 3.72	63.33 ± 1.53	108.48 ± 0.95		
33	Butanal		17 [31]	0.98 ± 0.12	1.94 ± 0.31	1.36 ± 0.23	1.00 ± 0.18	2.16 ± 0.11	0.000	1.29
34	3-Methylbutanal	AADV	0.2 [31]	0.87 ± 0.24	2.62 ± 0.49	1.74 ± 0.13	1.13 ± 0.14	3.54 ± 0.60	0.000	1.25
35	2-Methylbutanal	AADV	1 [31]	0.21 ± 0.08	3.70 ± 0.45	3.49 ± 0.47	2.29 ± 0.27	7.91 ± 0.78	0.000	1.14
36	Pentanal	FADV	12 [31]	0.28 ± 0.03	0.33 ± 0.03	0.17 ± 0.02	0.27 ± 0.06	0.33 ± 0.10	0.027	1.21
37	Hexanal	FADV	4.5 [10,33]	8.82 ± 1.45	4.41 ± 0.50	3.40 ± 0.45	2.71 ± 0.23	3.14 ± 0.38	0.000	0.84
38	(E)-2-hexenal	FADV	17 [8,15]	1.07 ± 0.07	3.40 ± 0.36	3.42 ± 0.48	4.15 ± 0.66	5.65 ± 0.34	0.000	0.93
39	Heptanal	FADV	2.8 [33]	0.30 ± 0.04	0.64 ± 0.08	1.16 ± 0.14	0.88 ± 0.12	2.18 ± 0.11	0.000	1.07
40	(E)-2-Heptenal	FADV	13 [31]	0.17 ± 0.03	0.24 ± 0.03	0.15 ± 0.02	0.18 ± 0.03	0.24 ± 0.04	0.017	1.16
41	Benzaldehyde	AADV	350 [33]	4.10 ± 0.62	4.62 ± 0.22	3.68 ± 0.24	6.26 ± 0.61	13.14 ± 1.13	0.000	1.17
42	(E,E)-2,4-Heptadienal	FADV	56 [31]	2.55 ± 0.77	2.04 ± 0.22	1.42 ± 0.06	1.60 ± 0.19	2.56 ± 0.57	0.031	0.97
43	Benzeneacetaldehyde	AADV	4 [3,8,31]	4.17 ± 0.99	9.72 ± 0.79	9.28 ± 0.99	15.71 ± 0.98	23.21 ± 2.21	0.000	1.07
44	2,6-Dimethyl-5-hepten-1-al			0.68 ± 0.20	0.69 ± 0.14	0.55 ± 0.06	nd	nd	0.441	0.62
45	(E)-2-Octenal	FADV	3 [10]	3.37 ± 0.25	2.01 ± 0.31	1.32 ± 0.11	1.61 ± 0.16	1.78 ± 0.12	0.000	0.92
46	Nonanal	FADV	1 [10]	13.89 ± 1.83	6.28 ± 1.05	6.87 ± 0.71	6.65 ± 0.76	15.65 ± 1.77	0.000	1.09
47	(E)-2-Nonenal	FADV	0.08 [15]	1.93 ± 0.42	1.37 ± 0.11	0.88 ± 0.19	1.11 ± 0.05	1.19 ± 0.30	0.005	0.88
48	Safranal	CDV	3 [33]	2.00 ± 0.39	2.21 ± 0.09	3.18 ± 0.24	3.02 ± 0.32	8.43 ± 0.70	0.000	1.12
49	Decanal	FADV	6 [15]	0.92 ± 0.34	2.44 ± 0.10	2.71 ± 0.22	2.61 ± 0.08	2.71 ± 0.21	0.000	0.86
50	(E,E)-2,4-Nonadienal	FADV	0.02 [8]	1.92 ± 0.69	1.14 ± 0.22	1.05 ± 0.13	0.80 ± 0.12	1.28 ± 0.16	0.024	0.77
51	β-Cyclocitral	CDV	3 [33]	2.73 ± 0.14	1.81 ± 0.43	3.47 ± 0.07	2.60 ± 0.33	4.68 ± 0.43	0.000	1.03
52	Neral	VT	53 [33]	1.02 ± 0.19	2.59 ± 0.81	1.68 ± 0.07	2.35 ± 0.29	2.35 ± 0.09	0.004	0.96
53	Geranial	VT	32 [20]	7.21 ± 0.71	9.52 ± 1.22	6.02 ± 0.07	6.38 ± 0.61	6.35 ± 0.57	0.001	1.17
	Ketones			10.12 ± 1.26	12.22 ± 1.22	11.2 ± 0.6	11.35 ± 0.81	11.28 ± 1.30		
54	6-Methyl-5-heptene-2-one	CDV	50 [15,31]	0.41 ± 0.03	0.93 ± 0.07	1.06 ± 0.06	0.83 ± 0.14	0.87 ± 0.08	0.000	1.03
55	5-Ethyl-6-methyl-3-hepten-2-one			0.53 ± 0.16	1.08 ± 0.60	0.81 ± 0.12	1.07 ± 0.13	0.79 ± 0.11	0.198	0.71
56	d-Verbenone	VT		0.89 ± 0.34	1.77 ± 0.32	1.22 ± 0.15	1.27 ± 0.35	1.15 ± 0.23	0.041	0.88
57	β-Damascenone	CDV	0.002 [7]	0.79 ± 0.1	1.12 ± 0.25	1.14 ± 0.21	1.19 ± 0.32	0.94 ± 0.14	0.212	0.93
58	α-Ionone	CDV	0.4 [8]	1.70 ± 0.29	1.17 ± 0.11	0.82 ± 0.02	0.63 ± 0.15	0.74 ± 0.14	0.000	0.88
59	Geranylacetone	CDV	60 [15,33]	0.73 ± 0.13	2.22 ± 0.20	2.23 ± 0.25	2.33 ± 0.37	2.19 ± 0.27	0.000	0.82
60	β-Ionone	CDV	0.007 [7]	5.08 ± 0.58	3.93 ± 0.60	3.92 ± 0.45	4.04 ± 0.75	4.61 ± 0.61	0.151	0.68
	Esters			87.13 ± 9.65	134.82 ± 10.94	147.22 ± 3.69	126.51 ± 10.63	99.88 ± 7.98		
61	Vinyl hexanoate	FADV	5 [33]	0.63 ± 0.07	0.17 ± 0.02	0.24 ± 0.02	0.17 ± 0.01	0.26 ± 0.05	0.000	0.88
62	(E)-3-Hexenyl butyrate	FADV		1.32 ± 0.40	2.02 ± 0.17	2.59 ± 0.63	1.89 ± 0.60	0.67 ± 0.08	0.003	1.13
63	Methyl salicylate	AADV	40 [3,15]	75.47 ± 8.69	117.86 ± 8.34	133.83 ± 3.14	113.58 ± 8.45	91.66 ± 7.57	0.000	1.10
64	cis-3-Hexenyl isovalerate	FADV/AADV	0.13 [15]	2.83 ± 0.67	2.36 ± 0.43	2.63 ± 0.30	2.47 ± 0.47	2.57 ± 0.19	0.761	0.43
65	Methyl geranate	VT		3.27 ± 0.57	4.25 ± 0.73	2.62 ± 0.15	2.03 ± 0.34	2.61 ± 0.24	0.002	1.18
66	cis-3-Hexenyl hexanoate	FADV	16 [32]	1.25 ± 0.22	4.82 ± 0.87	2.93 ± 0.23	3.39 ± 0.56	1.09 ± 0.15	0.000	1.05
67	Cyclohexyl hexanoate	FADV		1.61 ± 0.34	1.76 ± 0.45	0.78 ± 0.10	1.21 ± 0.35	nd	0.028	1.05
68	(E)-2-Hexenyl caproate	FADV		0.76 ± 0.20	1.59 ± 0.36	1.60 ± 0.17	1.77 ± 0.24	1.02 ± 0.08	0.001	0.94
	Heterocyclic compounds			2.72 ± 0.27	0.87 ± 0.04	1.10 ± 0.12	0.69 ± 0.09	1.14 ± 0.13		
69	2-Ethylfuran		8000 [33]	0.43 ± 0.11	0.46 ± 0.02	0.61 ± 0.06	0.31 ± 0.06	0.42 ± 0.09	0.009	1.47
70	(E)-2-(2-Pentenyl)furan			2.28 ± 0.24	0.42 ± 0.03	0.49 ± 0.06	0.38 ± 0.02	0.72 ± 0.08	0.000	0.88
	Sulfur compounds			3.28 ± 0.78	7.97 ± 1.00	5.26 ± 0.87	4.96 ± 0.44	6.57 ± 0.50		
71	Dimethyl sulfide	AADV	1.1 [31]	3.28 ± 0.78	7.97 ± 1.00	5.26 ± 0.87	4.96 ± 0.44	6.57 ± 0.50	0.000	1.13
	Total			497.58 ± 38.91	690.51 ± 34.09	715.27 ± 25.98	678.23 ± 33.05	596.61 ± 14.73		

Note: the data are shown as mean ± SD (n = 3); nd means not detected; *p*-value was calculated by ANOVA; VIP was obtained from PLS-DA.

**Table 3:** Odor descriptions and odor active values (OAVs) of identified volatile compounds.

No.	Volatile Compound	Odor Description	OAV				
			ZYQ-1	ZYQ-2	ZYQ-3	ZYQ-4	ZYQ-5
1	Naphthalene ^I^	Pungent [32]	6.2	2.7	6.2	6.8	4.1
2	(E)-β-Ocimene ^II^	Warm, floral, herbal, sweet [34]	115.5	206.3	223.4	311.8	205.4
3	Ocimene ^II^	Herbal, green [15]		1.3	1.3	1.0	
4	δ-Cadinene ^II^	Herbal, woody [32]	1.0	3.5	2.2	3.7	3.0
5	cis-Linalool oxide (furanoid) ^II^	Fruity [15]			1.3	1.8	2.0
6	trans-Linalool oxide (furanoid) ^II^	Fruity, fresh [15]		1.8	3.3	3.2	4.0
7	Linalool ^II^	Floral, sweet [33]	284.6	278.4	403.6	586.9	510.7
8	Geraniol ^II^	Rose-like, sweet [7]	63.3	100.0	87.1	63.8	37.3
9	Nerolidol ^II^	Floral, woody [31]				1.3	1.4
10	3-Methylbutanal ^II^	Malt [31]	4.4	13.1	8.7	5.7	17.7
11	2-Methylbutanal ^II^	Cocoa, almond [31]		3.7	3.5	2.3	7.9
12	Hexanal ^I^	Green, leafy, grassy [10,33]	2.0				
13	Benzene acetaldehyde ^II^	Woody, sweet, honey [3,8,31]	1.0	2.4	2.3	3.9	5.8
14	(E)-2-Octenal ^I^	Fresh, cucumber-like, fatty, green [10]	1.1				
15	Nonanal ^I^	Waxy, fresh, orange-like [10]	13.9	6.3	6.9	6.7	15.6
16	(E)-2-Nonenal ^I^	Watermelon, cucumber-like [15]	24.1	17.2	11.0	13.9	14.8
17	Safranal ^II^	Woody, spicy, medicinal [33]			1.1	1.0	2.8
18	(E,E)-2,4-Nonadienal ^I^	Cucumber-like [8]	95.9	56.9	52.6	40.2	64.2
19	β-Cyclocitral ^II^	Herbal, clean, rose-like, fruity [33]			1.2	0.9	1.6
20	β-Damascenone ^II^	Fruity, apple-like [7]	392.7	558.3	569.2	593.7	469.0
21	α-Ionone ^II^	Woody, violet-like, floral [8,33]	4.2	2.9	2.0	1.6	1.8
22	β-Ionone ^II^	Woody, violet, floral [7,33]	725.5	561.1	560.0	576.8	657.9
23	Methyl salicylate ^I^	Vanilla flavor [3,15]	1.9	2.9	3.3	2.8	2.3
24	cis-3-Hexenyl isovalerate ^II^	Fresh, green [15]	21.7	18.2	20.2	19.0	19.8
25	Dimethyl sulfide ^II^	Cabbage, sulfur, corn, molasses [31]	3.0	7.2	4.8	4.5	6.0
	Sum		1762.1	1844.4	1975.2	2252.5	2055.1
	Group I		145.1	86.0	80.0	70.4	101.1
	Group II		1617.0	1758.3	1895.2	2182.1	1954.0
	Group II/Group I		11.1	20.4	23.7	31.0	19.3

Note: I means that this volatile compound has an adverse affect on black tea aroma, and II means that this volatile compound is beneficial for black tea aroma.

## Data Availability

Data is contained within the article and Supplementary Material.

## Supplementary Materials

The following supporting information can be downloaded at: https://www.mdpi.com/article/10.3390/foods12234368/s1, Figure S1: The average proportion (%) of different kinds of volatile compounds in black tea samples; Table S1: The changes in the flavor index of various kinds of volatile compounds; Table S2: The fold changes of the flavor index of various kinds of volatile compounds.
