# Supplementary material for: Difference in Aroma Components of Black Teas Processed on Different Dates in the Spring Season
Source: Foods. 2023 Dec 4;12(23):4368. doi: 10.3390/foods12234368 (PMC10706345; doi:10.3390/foods12234368)
Supplement: Supplementary file 1 [file foods-12-04368-s001.zip › foods-2726367-supplementary.pdf]

Difference in Aroma Components of Black Teas Processed on Different Dates in the Spring Season

Penghui Yu [1,†], Yingjie Huang [2,†], Ziyi Li [2], Xi Zhao [1], Hao Huang [1], Ni Zhong [1], Hongfa Zheng [1,*] and Qincao Chen [2,*]

[1] Tea Research Institute, Hunan Academy of Agricultural Sciences, Changsha 410125, China; yphuihui@163.com (P.Y.); lzzx_11280609@163.com (X.Z.); haohuang_08@163.com (H.H.); daren_ni@163.com (N.Z.)
[2] College of Agriculture, Jiangxi Agricultural University, Nanchang 330045, China; hyj605791@163.com (Y.H.); 18931077210@163.com (Z.L.)
* Correspondence: hncyszhf@hunaas.cn (H.Z.); chenqincao@jxau.edu.cn (Q.C.)
† These authors contributed equally to this work.

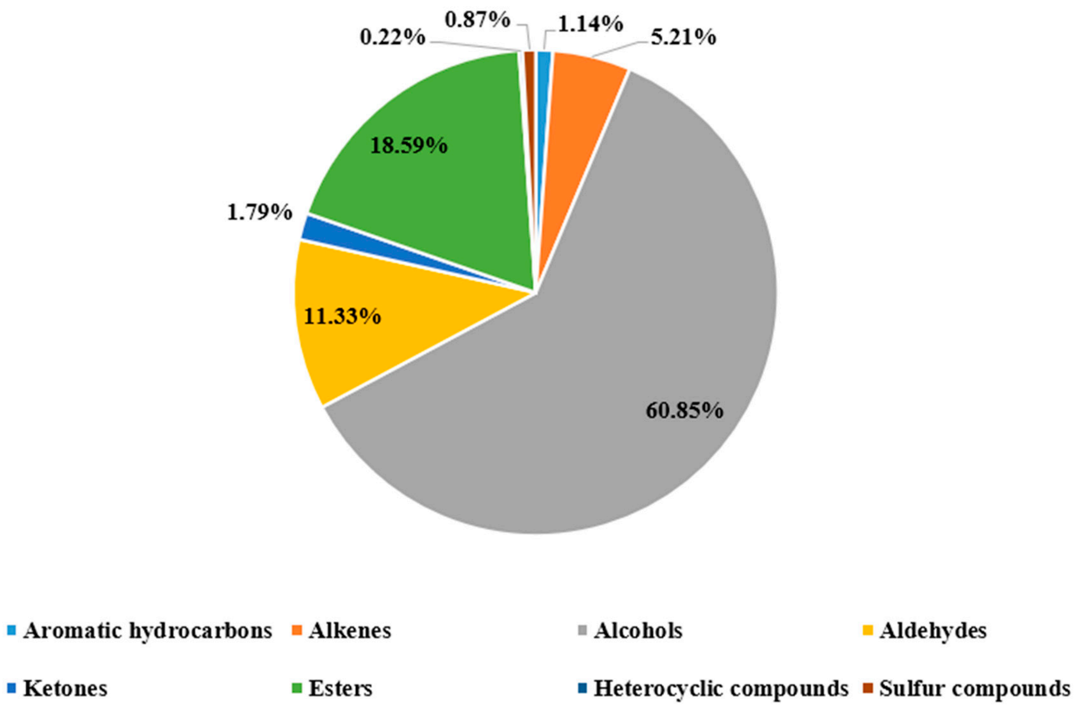

Figure S1. The average proportion (%) of different kinds of volatile compounds in black tea samples

Table S1. The changes in Flavor Index of various kinds of volatile compounds

	ZYQ-1	ZYQ-2	ZYQ-3	ZYQ-4	ZYQ-5
Flavor Index	11.66	23.25	18.86	17.58	11.02
VTs/non-ester FADVs	8.47	16.64	13.07	12.30	6.85
AADVs/non-ester FADVs	2.61	5.68	5.04	4.58	3.60
Ester FADVs/non-ester FADVs	0.22	0.45	0.30	0.30	0.11
CDVs/non-ester FADVs	0.35	0.48	0.45	0.41	0.46

Table S2. The fold changes of Flavor Index of various kinds of volatile compounds

	ZYQ-1	ZYQ-2	ZYQ-3	ZYQ-4	ZYQ-5
Flavor Index	1.00	1.99	1.62	1.51	0.95
VTs/non-ester FADVs	1.00	1.96	1.54	1.45	0.81
AADVs/non-ester FADVs	1.00	2.17	1.93	1.75	1.38
Ester FADVs/non-ester FADVs	1.00	2.05	1.37	1.37	0.52
CDVs/non-ester FADVs	1.00	1.35	1.26	1.15	1.38
